# The association between elder abuse and refrainment from daily activities during the COVID-19 pandemic among older adults in Japan: A cross-sectional study from the Japan Gerontological Evaluation Study

**DOI:** 10.1016/j.ssmph.2022.101229

**Published:** 2022-09-13

**Authors:** Chie Koga, Taishi Tsuji, Masamichi Hanazato, Koryu Sato, Katsunori Kondo

**Affiliations:** a Center for Preventive Medical Sciences, Chiba University, 1-33 Yayoi-cho, Inage-ku, Chiba-shi, Chiba, 263-8522, Japan; b Faculty of Health and Sport Sciences, University of Tsukuba, Tsukuba City, Japan; c Department of Social Epidemiology, Graduate School of Medicine and School of Public Health, Kyoto University, Yoshida-Konoecho, Sakyo-ku, Kyoto-shi, Kyoto, 606-8315, Japan; d Center for Gerontology and Social Science, National Center for Geriatrics and Gerontology, 7-430 Morikoka-cho, Obu-shi, Aichi, 474-8511, Japan

**Keywords:** Elder abuse, COVID-19, Behavioral refrain, Violence

## Abstract

**Objectives:**

Elder abuse is a public health issue that is thought to have increased during the COVID-19 pandemic due to lockdowns and behavioral restrictions. This study examines the association between elder abuse and refrainment from daily activities during the pandemic.

**Methods:**

We used data from a self-administered mail survey conducted by the Japan Gerontological Evaluation Study (JAGES) from November 2020 to February 2021 in 11 municipalities. Our participants included 18,263 older adults (age ≥65 years) who were independent in their daily lives. Logistic regression analysis was conducted to evaluate the association between elder abuse and refrainment from 10 daily activities, and the total number of refrained behaviors.

**Results:**

Experiences of abuse were reported by 288 participants (1.6%). The risk of elder abuse was 1.37 times (95% confidence interval, 1.04–1.81) higher among those who refrained from shopping for food and daily necessities and 1.60 times (1.20–2.13) higher among those who refrained from interaction with neighbors, than those who did not. Also, a dose-response relationship was observed where the risk of abuse increased with the number of restrictions.

**Conclusion:**

The risk of elder abuse increased as the number of refrained behaviors increased which suggests that refrainment from multiple behaviors may significantly increase the risk of elder abuse, compared with refrainment from a single behavior. To avoid increasing the risk of abuse in likely future pandemics, it is necessary to maintain social connections without face-to-face contact, or with adequate infection control measures.

## Highlights

• The risk of elder abuse was higher among those who refrained from shopping for food and daily necessities and from interaction with neighbors.
• The risk of abuse increased as the number of refrained behaviors increased.
• Our results illustrate that refraining from multiple behaviors may be significantly associated with an increased risk of abuse over that of refraining from a single behavior.

## 1 Introduction

The outbreak of the coronavirus pandemic (COVID-19) has restricted the activities of many people, including older adults (Lim et al., 2020). In March 2020, it was estimated that more than 3.4 billion people in 84 countries were restricted from going out (Bouziri et al., 2020). Although the Japanese Government did not implement lockdowns of cities or other legally-mandated measures, a stay-at-home order was complied with by the general population. In April 2020, major cities experienced 60%–80% reductions in the numbers of people going out. Home confinement raised concerns about the physical health of older adults, such as muscle weakness due to reduced physical activity, and about the effects on their psychological and social health, due to limited interactions with others (Maugeri et al., 2020; United Nations, 2020, pp. 1–16; Woods et al., 2020). There were also concerns about increasing violence, such as elder abuse in the home, due to lockdowns and restrictions on going out (Chang & Levy, 2021; Han & Mosqueda, 2020; Makaroun, Bachrach, & Rosland, 2020; United Nations, 2020, pp. 1–16).

Elder abuse is a public health challenge and is defined as “a single, or repeated act, or lack of appropriate action, occurring within any relationship where there is an expectation of trust, which causes harm or distress to an older person” by the World Health Organization (WHO, 2014). It reduces the well-being of older adults and negatively affects their health by increasing the risks of, for example, depression and suicide (Koga, Tsuji, Hanazato, Takasugi, & Kondo, 2022; Koga, Tsuji, Hanazato, Suzuki, & Kondo, 2020; Luo & Waite, 2011; Wong & Waite, 2017). Low level of social support and social capital can be risk factors for elder abuse (Johannesen & Logiudice, 2013; Koga, Hanazato, Tsuji, Suzuki, & Kondo, 2020; Park, 2018). Chang and colleagues studied 897 older adults in the United States and showed that elder abuse was positively associated with financial strain but negatively associated with a sense of community and physical distancing during the COVID-19 pandemic (Chang & Levy, 2021). Filipska and colleagues studied 347 hospitalized patients in Poland and found that low income, chronic diseases, poor relationships with family, and depression were associated with an increased risk of elder abuse during the pandemic (Filipska et al., 2021). Du and Chen recruited 10,362 Chinese older adults and reported that those who frequently participated in social activities were less likely to experience abuse during the pandemic (Du & Chen, 2021). This evidence indicated that social interaction is important in reducing the risk of elder abuse regardless of the COVID-19 pandemic. Although the association between social support and elder abuse has not been investigated enough, a study on 3157 older adults has reported that positive social interaction was a possible modifying effect on interpersonal strain (Zheng, Li, Kong, & Dong, 2019). Similarly, another study on 897 older adults argued that fewer interpersonal strains influenced mistreatment via loneliness, but social support moderated the influence. Moreover, the indirect effect of interpersonal strain on abuse via loneliness is more substantial for those with lower levels of support than those with higher levels of support (Chokkanathan, 2017). However, these studies during COVID-19 did not account for refrainment from various daily activities. Risk factors of elder abuse have been conceptualized in four categories: older person, perpetrator, their relationship, and the environment (Johannesen & Logiudice, 2013). Behavioral restrictions during the pandemic could affect relationship between older adults and caregivers and affect their surrounding environments. The risk of abuse may differ depending on the types of behaviors that are refrained from.

This study examines the association between elder abuse and refrainment from various behaviors in daily life during the COVID-19 pandemic. We used a large, representative dataset of older Japanese adults. We hypothesized that people who refrain from multiple behaviors are more likely to have an increased risk of elder abuse than those who refrain from a single behavior. By examining the relationship between refrainment from daily activities and elder abuse, this study can help prevent an increase in abuse during a future pandemic.

## 2 Materials and methods

### 2.1 Selection and description of participants and the setting

We used the cross-sectional data of a self-administered mail survey conducted by the Japan Gerontological Evaluation Study (JAGES) from November 2020 to February 2021 in 11 municipalities. We included questions on elder abuse to determine the frequency of elder abuse and its related factors during the COVID-19 pandemic. JAGES is a large, epidemiological study group of older adults (aged ≥65 years) throughout Japan who are without physical or cognitive disabilities (Kondo, Rosenberg, National Center for Geriatrics, & Gerontology, 2018). We randomly selected and sent questionnaires to 24,613 eligible people. We received responses from 19,928 participants (response rate, 81.0%). We excluded 1655 participants whose sex and age could not be confirmed or who were reported in error. Thus, the analytical population of this study was 18,263.

### 2.2 Elder abuse during the COVID-19 pandemic

The outcome variable was the presence or absence of elder abuse during the period of Japan's first declaration of a state of emergency (April to May 2020). Elder abuse was measured across three dimensions (physical, psychological, and financial) using a self-reported questionnaire developed in collaboration between researchers, doctors, social epidemiologists, and social workers (Koga, Hanazato, et al., 2020; Koga, Tsuji, et al., 2020). Because a definition of abuse has not been established, it is challenging to clarify criteria for determining what behavior constitutes elder abuse. To ascertain whether or not abuse had occurred, our study asked questions that referred to specific acts or feelings, such as being beaten, having one's self-esteem damaged, or having one's savings or pension taken. Participants were asked, “Please answer about your life during the period of the declaration of a state of emergency due to the outbreak of COVID-19 (April to May 2020). Please check all the boxes that apply to your daily activities.” For physical abuse, participants were asked, “Did you experience physical violence from your family, such as being hit, kicked, having objects thrown at you, or being shut in a room?” Psychological abuse was measured by the following question, “Did you experience an act by your family that harmed your self-esteem, such as verbal abuse, cutting remarks, or being ignored for long periods?” Financial abuse was measured by the question, “Do any of your family members take or use your savings or pension benefits without your consent?” Those who checked one or more of these questions were considered to have experienced abuse; other participants were deemed to not have experienced abuse.

### 2.3 Refrainment behaviors

The explanatory variables were the presence or absence of a decrease in 10 daily living behaviors and the total number of refrained behaviors (0, 1, 2, 3, or ≥4). Participants were asked, “Please answer about your daily life during the period of the declaration of a state of emergency (April to May 2020) due to the outbreak of COVID-19 infection. Please check all the boxes that apply to the activities that you reduced the frequency of or ceased: 1. Eating out; 2. Shopping for groceries and daily necessities; 3. Shopping for items other than groceries and daily necessities; 4. Exercising indoors, such as at a gym; 5. Exercising or walking outdoors; 6. Interacting with neighbors; 7. Visiting medical institutions (except in cases of suspected COVID-19 infection); 8. Using public transportation; 9. Visiting museums and movie theaters; and 10. Participating in community events. Each choice was recorded as 1, for those who decreased the frequency of the behavior or ceased it, or as 0, for those who did not change their behavior. The total number of refrained behaviors was the sum of refrained behaviors, excluding variables negatively associated with elder abuse. Those who refrained from four or more behaviors were merged into one category. This research is to determine the impact of COVID-19 on their lives and health of older adults ages 65 years and older who are not eligible for long-term care insurance. These options about activities in the daily lives of older adults were developed by the collective effort of researchers from various disciplines. Although the Japanese Government did not implement lockdown of cities or other legally mandated measures, people followed stay-at-home and refrained from their activity primarily by themselves.

### 2.4 Covariates

In line with former studies that focused on elder abuse, we included basic demographic information, including sex (male or female), age group (65–69, 70–74, 75–79, 80–84, and ≥85 years-old), educational attainment (≤9 or ≥10 years), employment status (never employed, past worker, or current worker), subjective socioeconomic status (poor, average, or rich), marital status (married, widowed, divorced, unmarried, or other), and living arrangements (alone, with family members, or other facilities), and change in income due to COVID-19 (decreased or not decreased) (Dong, Chen, Fulmer, & Simon, 2014; Dong, Simon, & Evans, 2014; Koga, Hanazato, et al., 2020; Koga, Tsuji, et al., 2020). Depressive symptoms were measured using the 15-item Geriatric Depression Scale, which defines mild and severe depression as 5–9 points and >10 points, respectively (Koga, Tsuji, et al., 2020). For measuring independence in activities of daily living, participants were asked, “Do you regularly receive nursing care or assistance for walking, bathing, and/or using a toilet?” Answers were recorded using the following 3-point scale: 1. No need for nursing care or assistance; 2. Requiring but not receiving nursing care or assistance; and 3. Needing and receiving nursing care or assistance.

### 2.5 Statistical analysis

Descriptive statistics were generated to summarize the characteristics of participants. To investigate the relationship between elder abuse and refrainment from behaviors during the COVID-19 pandemic, we conducted a logistic regression analysis and calculated the odds ratios (OR) and 95% confidence intervals (CI) for the risk of elder abuse, after adjusting for all covariates. We created two models. First, the 10 types of behavioral refrainment were simultaneously investigated in Model 1. In Model 2, the total number of refrained behaviors was added to Model 1. We calculated *p* for trend when the total number of refrained behaviors was included as a continuous variable. Because some variables in the analysis were missing, multiple imputations were performed. A total of 20 multiple imputed datasets were created, including all measured variables, using a multivariate normal imputation method with a “missing at random” assumption. The estimated parameters were combined using Rubin's combination methods (Rubin, 2004). All statistical analyses were conducted using Stata 16/IC (StataCorp, College Station, TX, USA).

## 3 Results

### 3.1 Participants’ characteristics

Table 1 shows the characteristics of study participants. The number of those who experienced abuse during the declared state of emergency was 288 (1.6%). Those who reduced their activities were 12680 (69.5%) for eating out, 3822 (21.0%) for shopping for groceries and daily necessities, 6694 (36.7%) for shopping for items other than groceries and daily necessities, 3875 (21.2%) for exercising indoors, such as at a gym, 2741 (15.0%) for exercising or walking outdoors, 4246 (23.3%) for interacting with neighbors, 3107 (17.0%) for visiting medical institutions (except in cases of suspected COVID-19 infection), 7321 (40.1%) for using public transportation, 7426 (40.7%) for visiting museums and movie theaters, and 5690 (31.2%) for participation in community events).

**Table 1:** Characteristics of study participants.

	All	%	Experienced abuse	%
Elder abuse	18236	100.0%	288	1.6%
Number of refrained behaviors				
None	2625	14.4%	22	0.8%
One decreased	2362	13.0%	27	1.1%
Two decreased	3023	16.6%	41	1.4%
Three decreased	2911	16.0%	47	1.6%
More than four	7315	40.1%	151	2.1%
Eating out				
Not decreased	5556	30.5%	93	1.7%
Decreased	12680	69.5%	195	1.5%
Shopping for groceries and daily necessities				
Not decreased	14414	79.0%	195	1.4%
Decreased	3822	21.0%	93	2.4%
Shopping for items other than groceries and daily necessities				
Not decreased	11542	63.3%	165	1.4%
Decreased	6694	36.7%	123	1.8%
Exercising indoors, such as at a gym				
Not decreased	14361	78.8%	209	1.5%
Decreased	3875	21.2%	79	2.0%
Exercising or walking outdoors				
Not decreased	15495	85.0%	216	1.4%
Decreased	2741	15.0%	72	2.6%
Interacting with neighbors				
Not decreased	13990	76.7%	177	1.3%
Decreased	4246	23.3%	111	2.6%
Visiting medical institutions (except in cases of suspected COVID-19 infection)				
Not decreased	15129	83.0%	216	1.4%
Decreased	3107	17.0%	72	2.3%
Using public transportation				
Not decreased	10915	59.9%	155	1.4%
Decreased	7321	40.1%	133	1.8%
Visiting museums and movie theaters				
Not decreased	10810	59.3%	153	1.4%
Decreased	7426	40.7%	135	1.8%
Participating in community events				
Not decreased	12546	68.8%	172	1.4%
Decreased	5690	31.2%	116	2.0%
Sex				
Male	8669	47.5%	106	1.2%
Female	9567	52.5%	182	1.9%
Age group				
65–59	3300	18.1%	54	1.6%
70–74	5483	30.1%	74	1.3%
75–79	4438	24.3%	67	1.5%
80–84	3113	17.1%	59	1.9%
85≤	1902	10.4%	34	1.8%
Education				
≤9 yrs	4310	23.6%	90	1.5%
≥10 yrs	13469	73.9%	188	1.2%
Missing	457	2.5%	10	1.0%
Employment status				
Never	2083	11.4%	34	1.6%
Past worker	10239	56.1%	163	1.6%
Current worker	5306	29.1%	81	1.5%
Missing	608	3.3%	10	1.6%
Subjective socioeconomic status				
Poor	3982	21.8%	141	3.5%
Usual	10759	59.0%	114	1.1%
Rich	3228	17.7%	29	0.9%
Missing	267	1.5%	4	1.5%
Marital status				
Married	12246	67.2%	180	1.5%
Widowed	3589	19.7%	54	1.5%
Separated	1029	5.6%	27	2.6%
Unmarried	788	4.3%	18	2.3%
Other	123	0.7%	5	4.1%
Missing	461	2.5%	4	0.9%
Living arrangement				
Living alone	3443	18.9%	66	1.9%
Living with someone	14618	80.2%	219	1.5%
Missing	175	1.0%	3	1.7%
Depression				
Normal	12607	69.1%	99	0.8%
Mild depressive	3626	19.9%	104	2.9%
Severe depressive	1214	6.7%	72	5.9%
Missing	789	4.3%	13	1.6%
Change in income due to COVID-19				
Decreased	2213	12.1%	56	2.5%
Not decreased	15100	82.8%	215	1.4%
Missing	923	5.1%	17	1.8%
Activity of Daily Living (ADL)				
Unnecessary	16263	89.2%	212	1.3%
Necessary and supported	942	5.2%	38	4.0%
Necessary and not supported	543	3.0%	25	4.6%
Missing	488	2.7%	13	2.7%

Not experienced abuse, n = 17,948 (98.4%); Experienced abuse, n = 288 (1.6%).

### 3.2 Results of logistic regression analysis

Table 2 shows the OR for the associations between elder abuse and behavioral refrainment among older Japanese adults. In Model 1, the risks of abuse were 1.37 times (CI, 1.04–1.81) and 1.60 times (CI, 1.20–2.13) higher, respectively, for those who reduced grocery shopping and interaction with neighbors. than for those who did not. In contrast, those who reduced eating out had a 0.75 times (CI, 0.54–0.96) lower risk of abuse than those who did not. Because the variable *eating out* was negatively associated with abuse in Model 1, it was excluded from Model 2, along with the total number of refrained behaviors. In Model 2, the risk of abuse was 1.84 times (CI, 1.09–1.82) higher for two reduced behaviors, 2.40 times (CI, 1.31–4.41) higher for three reduced behaviors, and 2.86 times (CI, 1.25–6.54) higher for four or more reduced behaviors, compared with those who had zero reduced behaviors (excluding the *eating out* variable). When the total number of refrained behaviors was treated as a continuous variable, its *p* for trend was 0.003. For sensitivity analysis, we included the *eating out* variable in Model 2, and found a similar result (Supplementary Table).

**Table 2:** Odds ratios for the association between elder abuse and behavioral decline among older Japanese adults (n = 18,236).

	Model 1				Model 2			
	ORs	p	95% CI		ORs	p	95% CI	
Number of refrained behavior								
None					1.00			
One decrease					1.12	0.666	0.68	1.84
Two decrease					1.84	0.022	1.09	3.10
Three decrease					2.40	0.005	1.31	4.41
More than four					2.86	0.013	1.25	6.54
Eating out								
Not decrease	1.00							
Decreased	0.75	0.049	0.56	1.00				
Shopping for groceries and daily necessities								
Not decrease	1.00				1.00			
Decreased	1.37	0.027	1.04	1.81	1.16	0.319	0.86	1.57
Shopping for items other than groceries and daily necessities								
Not decrease	1.00				1.00			
Decreased	0.91	0.520	0.69	1.20	0.74	0.047	0.55	1.00
Exercising indoors, such as at a gym								
Not decrease	1.00				1.00			
Decreased	1.15	0.383	0.84	1.57	1.05	0.744	0.77	1.44
Exercising or walking outdoors								
Not decrease	1.00				1.00			
Decreased	1.16	0.327	0.86	1.57	1.01	0.952	0.74	1.38
Interacting with neighbors								
Not decrease	1.00				1.00			
Decreased	1.60	0.001	1.20	2.13	1.35	0.052	1.00	1.82
Visiting medical institutions (except in cases of suspected COVID-19 infection)								
Not decrease	1.00				1.00			
Decreased	1.22	0.171	0.92	1.64	1.06	0.696	0.78	1.44
Using public transportation								
Not decrease	1.00				1.00			
Decreased	1.11	0.455	0.84	1.46	0.89	0.425	0.66	1.19
Visiting museums and movie theaters								
Not decrease	1.00				1.00			
Decreased	1.23	0.208	0.89	1.70	0.96	0.802	0.68	1.35
Participation in community events								
Not decrease	1.00				1.00			
Decreased	1.16	0.366	0.84	1.60	0.99	0.934	0.71	1.37

n = 18,236. Model 1 and Model 2 adjusted for age, sex, education attainment, employment status, subjective socioeconomic status, marital status, living arrangement, depression, change in income, and activity of daily living.

## 4 Discussion/conclusion

This investigation revealed that the risk of elder abuse was higher among those who refrained from shopping for food and daily necessities and from interaction with neighbors. The risk of abuse increased as the number of refrained behaviors increased. Our results demonstrated that refrainment from multiple behaviors might be significantly associated with an increased risk of abuse over that of refrainment from a single behavior. To the best of our knowledge, this is the first study to investigate the association between elder abuse and refrainment from various daily activities during the COVID-19 pandemic.

We found clear associations between abuse and refrainment from shopping for food and daily necessities and from interaction with neighbors. These two activities can be considered the most routinely performed among the explanatory variables. In Japan, there was no substantial restriction on going out, such as a lockdown. However, the government recommended that people avoid places that meet the 3 Cs (closed space, crowded place, and close-contact setting) (Lim et al., 2020). People and communities that faithfully followed the instructions might have had a higher risk of abuse. Restriction of activities, such as shopping and social interaction, could be an indicator of increased risk of abuse. Previous studies have suggested that social support can be a protective factor against elder abuse (Chokkanathan, 2017; Dong & Simon, 2008; Melchiorre et al., 2013). Therefore, proactive contact (e.g., from neighbors) with older adults who are no longer seen in neighborhoods (e.g., at the supermarket) may help prevent abuse.

This study also showed that the risk of elder abuse increased as the number of refrained behaviors increased. Previous studies have reported that abuse occurs most commonly among family members living together (Takasaki et al., 2005). When behaviors in daily life are restricted and the time spent at home is increased, more opportunities arise for conflicts among family members living together, contributing to an increased risk of abuse. Although our findings did not reach significance level for some of the refrained behaviors, their scores indicated a trend toward increasing risk. In other words, they suggested that refrainment from multiple behaviors in daily life could accumulate a substantial risk.

Contrary to our hypothesis, we found that refrainment from eating out was negatively associated with the risk of elder abuse. This finding is consistent with a previous study, which suggested that maintaining physical distance (staying six feet apart from other persons) was associated with a lower risk of abuse during the pandemic (Chang & Levy, 2021). Out of our participants, 69.5% answered that they had reduced the frequency of eating out, which was the most common behavioral refrainment. It is possible that those who reduced eating out also followed social norms and considered social benefits. Such people may tend to have strong social ties, which can mitigate the risk of abuse (Webster et al., 2020).

Based on these results, policy recommendations would be as follows. Increase the home visiting and watching over towards older adults who decrease to go out. Moreover, actively promote the use of appropriate services is important for families who are considered to have a heavy burden of care.

### 4.1 Strengths and limitations

This study explored the associations between elder abuse and refrainment from various behaviors in daily life during the COVID-19 epidemic. We found the risk of abuse to be differentiated by the type of refrained behavior. We used a large, representative dataset that was collected using rigorous random sampling methods; many earlier studies related to the COVID-19 pandemic have relied on online surveys or convenience sampling. However, several limitations of our study must be mentioned. First, there is a possibility of reverse causality due to the cross-sectional design. Those who experienced abuse may have been restricted in their daily lives by their abusers. Therefore, future longitudinal studies should be conducted. Second, there is a possibility of selection bias, due to nonrespondents. Participants who suffered from severe abuse may not have responded to the questionnaire. It should be noted also that we studied only older adults who were physically and cognitively independent. Despite the fact that physical and cognitive impairments are significant risk factors of abuse, we could not invite such participants (Du & Chen, 2021; Filipska et al., 2021). Third, our study recruited only Japanese participants. Therefore, generalizability to other countries with different pandemic situations and cultures should be verified. Thus, we cannot discuss any differences related to cultural contexts. Fourth, reporting errors in the presence of abuse may have occurred; our questionnaires were self-reported and have not been validated. The observed prevalence of abuse (1.6%) may seem very low, given that a previous meta-analysis estimated the global prevalence to be 15.7% (Yon, Mikton, Gassoumis, & Wilber, 2017). Nonetheless, the rate we observed was reasonable. In 2019 in Japan, 34,057 cases were consulted or reported to municipalities; 16,928 cases were confirmed as elder abuse by caregivers in an older population of approximately 36 million (Ministry of Health Labor and Welfares of Japan, 2019). Despite the above limitations, this study has provided important perspectives regarding the relationship between elder abuse and the reduction of behaviors during the COVID-19 pandemic.

## 5 Conclusions

The risk of elder abuse was higher among those who refrained from shopping for food and daily necessities and from interaction with neighbors than among those who did not. The risk of abuse increased as the number of refrained behaviors increased. This indicates that refrainment from multiple behaviors may significantly increase the risk of elder abuse above that of refrainment from a single behavior. Because another pandemic is likely to occur in the future, it is necessary to maintain social connections, without face-to-face contact or with adequate infection control measures, to avoid increasing the risk of abuse.

## Statement of ethics

This study was reviewed and approved by the ethics committees of Chiba University (3442). The study was conducted in accordance with the principles of the Declaration of Helsinki and its later amendments. All participants provided written informed consent when they returned a questionnaire.

## Funding sources

This study used data from Longevity Science Research and Development Project, 10.13039/100009619Japan Agency for Medical Development (AMED) (JP19dk0110034, JP20dk0110034), Social welfare and epidemiological research for planning measures to cope with lifestyle issues caused by socioeconomic disparity at the National Institute for Longevity Sciences (H30-Cardiovascular, etc.-General-004), 10.13039/501100001691JSPS (Japan Society for the Promotion of Science) KAKENHI (grant Nos. 20K13721), a JST-OPERA program grant (JPMJOP1831), and costs commissioned by municipalities.

## Author contributions

C.K.: conceived the design, analyzed the data, and wrote the first draft of the article. C.K. and K.S.: performed the literature review. T.T., K.S., and K.K.: collected the data. K.K. is the principal.

## Declaration of competing interest

The authors have no conflicts of interest to declare.

## Data Availability

Data will be made available on request.

## Glossary

OR: odds ratio
CI: confidence interval
