# Supplementary material for: The association between elder abuse and refrainment from daily activities during the COVID-19 pandemic among older adults in Japan: A cross-sectional study from the Japan Gerontological Evaluation Study
Source: SSM Popul Health. 2022 Sep 13;19:101229. doi: 10.1016/j.ssmph.2022.101229 (PMC9467916; doi:10.1016/j.ssmph.2022.101229)
Supplement: Multimedia component 1 [file mmc1.docx]

**Supplementary Table.** Odds ratios for Model 2, including the variable “eating out” in the number of refrained behaviors

|  | Model 2 | | | |
| --- | --- | --- | --- | --- |
|  | ORs | p | 95% CI | |
| Number of refrained behavior (Include eating out variable) | | | | |
| None | 1.00 |  |  |  |
| One decrease | 1.74 | 0.064 | 0.97 | 3.15 |
| Two decrease | 2.34 | 0.004 | 1.31 | 4.20 |
| Three decrease | 2.97 | 0.001 | 1.59 | 5.56 |
| More than four | 3.19 | 0.003 | 1.48 | 6.88 |
| Eating out |  |  |  |  |
| Not decrease | 1.00 |  |  |  |
| Decreased | 0.57 | 0.000 | 0.42 | 0.78 |
| Shopping for groceries and daily necessities | | | |  |
| Not decrease | 1.00 |  |  |  |
| Decreased | 1.21 | 0.184 | 0.91 | 1.62 |
| Shopping for items other than groceries and daily necessities | | | | |
| Not decrease | 1.00 |  |  |  |
| Decreased | 0.83 | 0.204 | 0.63 | 1.10 |
| Exercising indoors, such as at a gym | | |  |  |
| Not decrease | 1.00 |  |  |  |
| Decreased | 1.12 | 0.453 | 0.83 | 1.53 |
| Exercising or walking outdoors | |  |  |  |
| Not decrease | 1.00 |  |  |  |
| Decreased | 1.07 | 0.669 | 0.79 | 1.45 |
| Interacting with neighbors | |  |  |  |
| Not decrease | 1.00 |  |  |  |
| Decreased | 1.47 | 0.009 | 1.10 | 1.97 |
| Visiting medical institutions (except in cases of suspected COVID-19 infection) | | | | |
| Not decrease | 1.00 |  |  |  |
| Decreased | 1.12 | 0.441 | 0.84 | 1.51 |
| Using public transportation | |  |  |  |
| Not decrease | 1.00 |  |  |  |
| Decreased | 0.98 | 0.901 | 0.74 | 1.30 |
| Visiting museums and movie theaters | | |  |  |
| Not decrease | 1.00 |  |  |  |
| Decreased | 1.09 | 0.622 | 0.78 | 1.51 |
| Participation in community events | | |  |  |
| Not decrease | 1.00 |  |  |  |
| Decreased | 1.08 | 0.618 | 0.79 | 1.49 |

**Abbreviations:** OR, odds ratio; CI, confidence interval.
